# The Altar Machine in the Church Mother of Gangi (Palermo, Italy). Interpretation of the past uses, scientific investigation and preservation challenge

**DOI:** 10.1186/1752-153X-6-47

**Published:** 2012-05-22

**Authors:** Angela Lo Monaco, Maurizio Marabelli, Claudia Pelosi, Michele Salvo

**Affiliations:** 1 Department of Agriculture, Forests, Nature and Energy, Tuscia University, Viterbo, Italy; 2 Department of Cultural Heritage Sciences, Tuscia University, Viterbo, Italy

**Keywords:** Altar Machine, Wood, Painting materials, State of preservation

## Abstract

**Background:**

The aim of this work was to study the Altar Machine in the Church Mother of Gangi, a little town near Palermo (Italy) regarding the history, the technical manufacture, the constitutive materials and the state of preservation.

The Altar Machine was dated back to the second half of the 18^th^ century; it is constituted by carved and painted wood, a complex system of winch and pulleys allows move various statues and parts of the Machine in accordance with the baroque scenography machineries.

**Results:**

The observation and survey of the mechanisms allowed formulate hypothesis on a more ancient mode of operation of the Altar Machine.

Laboratory analysis revealed the presence of many superimposed layers constituted by several different materials (protein binders, siccative oils, natural terpene resins, shellac, calcium carbonate, gypsum, lead white, brass, zinc white, iron oxides) and different wood species employed for the original and restoration elements of the Machine. This is due to a continuous usage of the object that has got a demo-ethno-anthropological significance.

Microclimate monitoring (relative humidity RH and temperature T) put in evidence that most of the data fall outside the tolerance intervals, i.e. the RH and T limits defined by the international standards. In particular, T values were generally high (out of the tolerance range) but they appeared to be quite constant; on the other hand RH values fell almost always inside the tolerance area but they often exhibited dangerous variations.

**Conclusions:**

The characterization of the constitutive materials provided useful information both to support the dating of the Machine proposed by the inscription and to obtain a base of data for a possible conservation work.

The microclimate monitoring put in evidence that the temperature and relative humidity values are not always suitable to correctly preserve the artefact. The careful in situ investigation confirmed an on-going climate induced damage to the Altar Machine that, associated to the deterioration caused by its usage, may have dramatic consequences on this unique and peculiar work of art.

The results of this work will have potential implications in the near future regarding a probable conservation project on the Machine.

## Background

**Figure 1:**
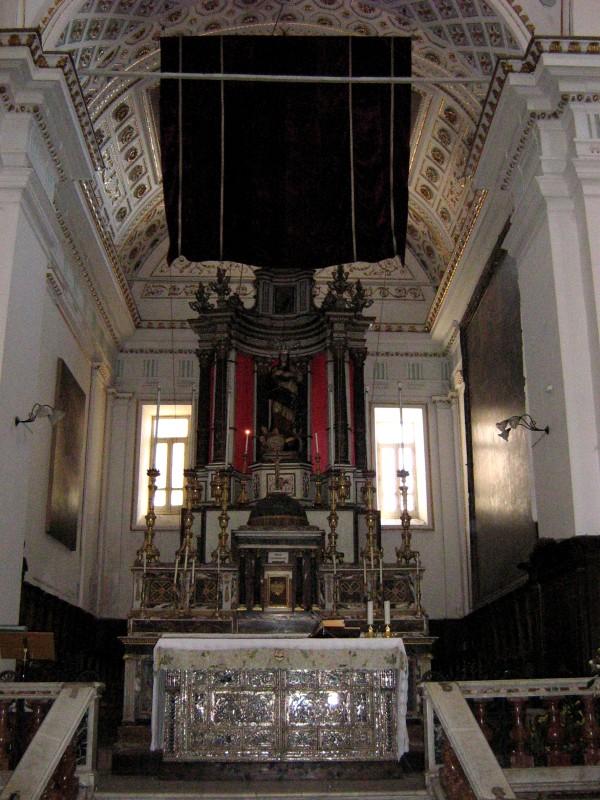
A view of the main altar of the Church Mother in Gangi (Italy).

The Altar Machine, object of this paper, was dated back to the second half of the 18^th^ century and it was attributed to the sculptor Fabio Pane (Figure 1). It is located in the Church Mother of Gangi (Palermo, Italy) whose first nucleus was built in the 16^th^ century. The Church Mother was consecrated to *San Nicola di Bari*; in fact, a statue of this saint is stored in the apse, behind the Machine. The main information about the Church Mother and the Altar Machine has been derived from the unpublished documents stored in the archive of the church [1] and from poor literature references [2]. 

The Altar Machine is a particular and complex system about 8,35 meters high. As we will see later, the different parts of the machine are made of various wood species.

**Figure 2:**
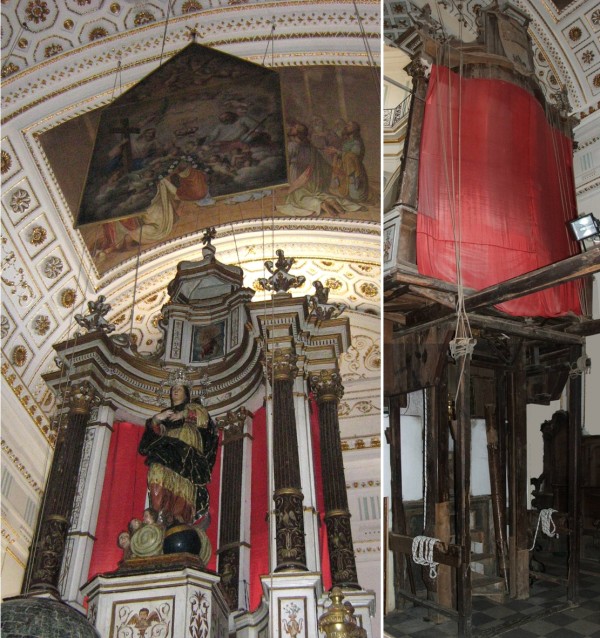
**A general view of the Altar Machine, front and back.** The Holy Trinity painting is also visible.

The upper and frontal part of the altar is carved and painted whereas the back side shows the bare wood (Figure 2). The lower part of the machine, hidden by the marble altar and by the *predella*, has a bearing function and contains a winch connected to some pulleys by means of ropes. This winch has the function to move the Altar Machine allowing put on the Virgin’s Apotheosis in the evenings of 14 and 15 of August. This event is named by the local people with the dialect expression: “*acchianata da Madonna*” (the Virgin’s Ascent) [3]. In particular, during the August celebrations, the machine movement causes the ascent of the Virgin’s statue and simultaneously the descent of a painting that portrays the Holy Trinity while crowning the Virgin (Figure 2). When the statue and the painting meet – the silver crown of the statue fills the cavity visible on the painting - the painting is suddenly raised and it disappears behind a red velvet curtain. 

The frontal part of the Altar Machine is characterized by some architectural elements that must be briefly described. Four angular plinths rest on the top of the framework (Figure 2). From the plinths a curved stylobate leads off. Four columns are based on the plinths, whereas two pilasters rest on the stylobate. The architectural elements have a composite order and they appear cabled with zoomorphic motifs. The intercolumniation is hidden by a red curtain.

The trabeation is decorated with winged puttos and it is surmounted by a cyma with the respective removable canopy and the monstrance. At the ends of the trabeation, two couples of ornamental vases can be observed. The cyma frames an icon representing the sinner angel expelled from the heaven.

**Figure 3:**
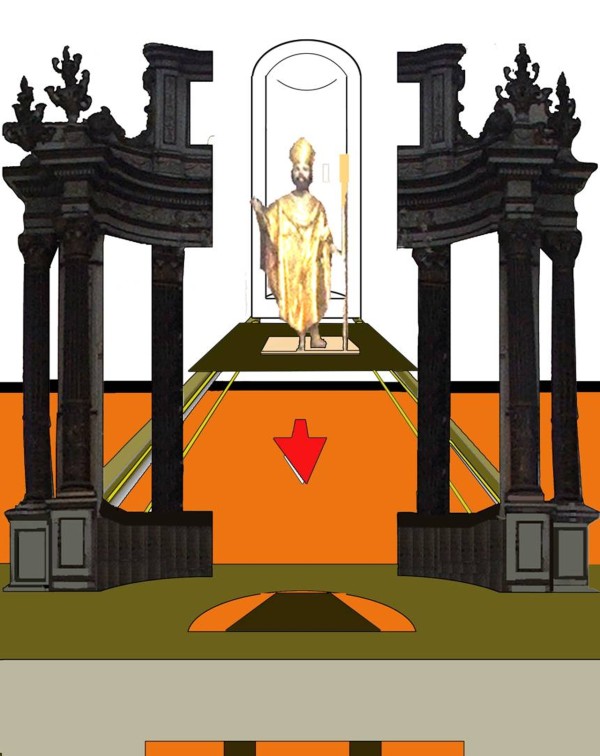
A virtual reconstruction of the Machine with the supposed opening and the San Nicola statue that moves forward.

On the back of the altar, two wooden beams (partially visible in Figure 2), fasten the Machine to the apse wall. Two pulleys, put at the end of the beams, probably had also the function to move the statue of *San Nicola di Bari*. The presence of the saint statue together with two bolts in the upper and lower part of the Machine allowed us hypothesize that probably the Machine opened enabling the *San Nicola* statue to move forward (Figure 3).

**Figure 4:**
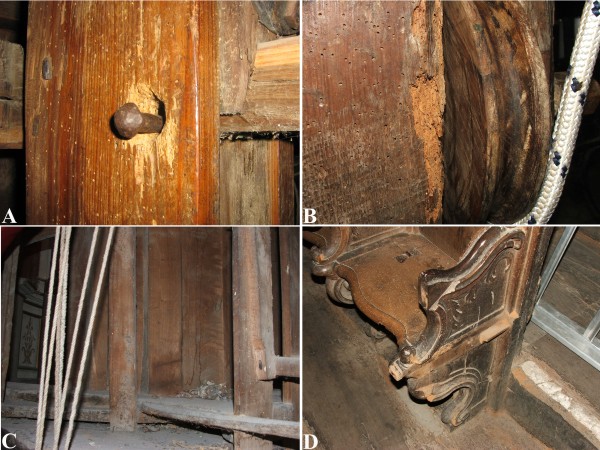
**Some details of the damages in the Altar Machine.A**) xylophages attack and scratches due to the use; **B**) xylophages attack and wood erosion; **C**) various kinds of stains and dirt layers; **D**) xylophages attack in the choir that encircles the Altar.

The carefully done in situ observation of the Altar Machine allowed detect several damages on the frontal polychrome surface, like cracks and detachments of the painted layers, whereas on the back side the wood elements are visible as they are not covered by any paint. So, the original wood defects were identified like knots, ring shake, etc. Moreover, wood deterioration was observed due to the wood manufacturing, to the Altar Machine usage, and to the microclimate of the church. Several scratches, dust, wood deformations, xylophages attack, and wood colour changes were observed (Figure 4).

It is interesting to note that on the stylobate there is an inscription “A. M. FABIO PANE EXTRUCTER – ARCH. JOSEPHO BLASCO – SAC. EPIFHANIO ANDALORO DEPICTA FUI AN. 1908”, that allows to state that the Altar Machine was restored at the beginning of the 20^th^ century but it was created by Fabio Pane, a woodcarver born in 1738, according to the baptism book in the Parish Archive of the Church Mother. On the basis of this inscription, the Altar Machine creation can be attributed to Fabio Pane.

The aim of this work has been to evaluate the state of preservation of the artefact and to investigate the materials both to know the realization technique and to obtain useful data in anticipation of a possible conservation project which at present is under evaluation. As support to the project a short but significant microclimatic campaign was realized [4].

The Machine is a particularly delicate system made of several materials: wood, pigments, binders, gilding, so the study and characterization of the constitutive materials required different laboratory techniques. We think that the scientific investigations on objects that have both an historical artistic and ethno anthropological value could supply a valid aid to a better comprehension of their usage and of their significance for the peoples [5], to date them in case of uncertain chronological attribution [6] and to have useful information to support the conservation work.

Regarding the microclimatic data we considered the so called tolerance intervals [7]. For wood and painted wooden sculptures this area has been set between 19 and 24°C as regards temperature, with a tolerance of 1,5°C, and between 50 and 60% as regards relative humidity (RH%), with a tolerance of 4%. Daily temperature and moisture cycles cause mechanical stress in wooden artefacts [8,9] that can affect also the painted layers. Moreover, under specific conditions, they also make wood susceptible to biotic degradation. Therefore a microclimate campaign, even if it was carried out for a short period, appeared useful to evaluate if the thermo hygrometric parameters of the Machine environment were included within the tolerance range and if they could be suitable for the conservation of the artefact. [10].

## Experimental

Microsamples taken from representative areas of the object were analysed (Table 1).

**Table 1:** Sample description and their location on the Altar Machine

**Sample**	**Location**	**Description**
ASN1	Front, white area of the frame	White area
ASN2	Right side, brown area of the floral element	Brown area
ASN3	Right side, grey area with traces of gilding	Gilding
ASN4	Right side, brown area at the base of the column	Brown area
ASN5	Front, brown area of the frame with traces of blue colour	Brown area
ASN6	Altar decoration	Wood
ASN7	Pulley	Wood
ASN8	Framework	Wood
ASN9	Winch drum	Wood
ASN10	Tilting table	Wood
ASN11	Tabernacle	Wood

A small quantity of samples ASN1, ASN2, ASN4 and ASN5 was mounted in polyester transparent resin. Polished cross-sections were prepared from the samples according to traditional techniques. Observation and photography of the sample cross-sections were performed by a Zeiss Axioskop polarizing microscope equipped with a Zeiss AxioCam digital camera. Cross-sections were studied also under UV lighting using a Mercury Vapour lamp directly connected to the microscope in order to observe fluorescence of the materials. A filter was interposed between the mercury lamp and the sample with the following characteristics: excitation BP 365/12, beamsplitter FT 395, and emission LP 397.

Infrared spectra were obtained using a Nicolet Avatar 360 Fourier transform spectrometer. For each sample 128 scans were recorded in the 4000 to 400 cm^-1^ spectral range in diffuse reflection modality (DRIFT) with a resolution of 4 cm^-1^. Spectral data were collected with OMNIC 8.0 (Thermo Electron Corporation) software. Samples were ground with spectrophotometric grade KBr (1% sample in KBr) in an agate mortar. As background the spectrum of the KBr powder has been used.

Samples were also examined by X-Ray fluorescence spectroscopy by means of a Surface Monitor instrument supplied by Assing. The XRF spectra were obtained with the following experimental conditions: Mo tube operating at 25 kV voltage and 300 μA beam current; scan time 120 s; distance 95 mm.

Regarding wood samples, thin sections were obtained according to the anatomic wood directions [11] and described following the IAWA list of microscopic features for hardwood and softwood identification [12,13]. Thin sections of the wood sample were examined under a Polyvar 100 optical microscope equipped with a PIXeLINK digital camera.

Temperature (T) and relative humidity (RH) values have been recorded through a digital data logger Testo 177-H1 model. The data logger has been calibrated and hanged on the northern wall of presbytery. Data have been recorded from 26 of July to 8 of September and then elaborated by Excel software to obtain maximum, minimum, average values and standard deviation throughout the analysed period. The temperature and relative humidity performance and failure indexes were also calculated and showed in the tolerance matrix, in order to obtain a synthetic expression of the results [14,15].

## Results and discussion

### Painting material analysis

**Figure 5:**
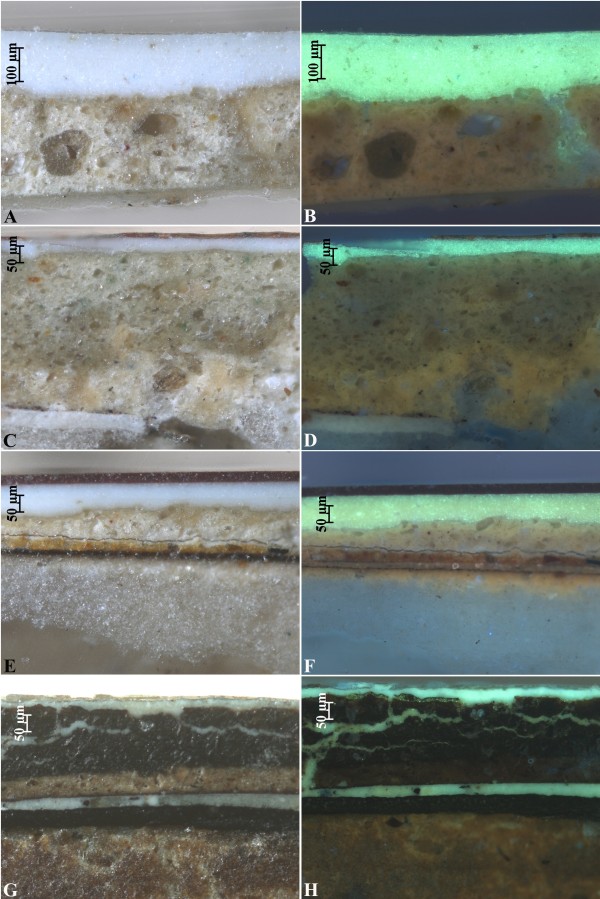
Microphotographs of samples ASN1 (A, B), ASN2 (C, D), ASN4 (E, F) and ASN5 (G, H), under reflected light (A, C, E, G) and UV fluorescence (B, D, F, H).

Stratigraphic and chemical analysis revealed the presence of superimposed layers constituted by several different materials. For this kind of artefacts it is usual to find many superimposed painted layers due to a continuous usage of the object that has got a demo-ethno anthropological significance. In Figure 5 the cross sections of the painting samples are showed. The white layer characterized by an intense yellow fluorescence is constituted by zinc white a pigment widely used starting from 19^th^ century. According to this result it is possible to assess that the surface painted layer was certainly applied during the 19^th^ or later. The painted layers were applied over gypsum and glue, as revealed by FTIR analysis and UV fluorescence examination of the cross sections. Glue exhibits a light blue fluorescence under UV lighting. At last, in sample ASN2 and ASN4 an orange UV fluorescence can be observed. This fluorescence can be associated to the presence of shellac, a natural resin often used with the function to isolate the priming or the support before applying the painted layers or the setting respectively.

**Figure 6:**
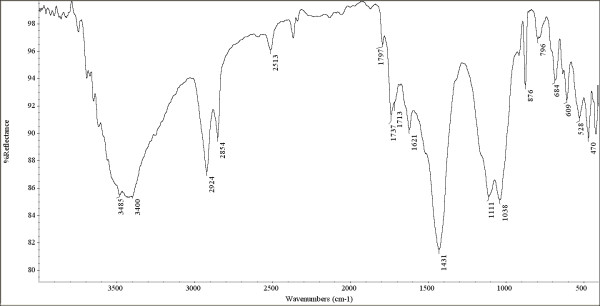
FTIR spectrum of sample ASN1.

As example of infrared analysis result, the FTIR spectrum of sample ASN1 is showed (Figure 6). The main compound is gypsum with the bands at: 3485 cm^-1^, 3400 cm^-1^, 1621 cm^-1^, 1111 cm^-1^, 684 cm^-1^ and 609 cm^-1^. Moreover, calcium carbonate (bands at: 2513 cm^-1^, 1797 cm^-1^, 1431 cm^-1^ and 875 cm^-1^), a siccative oil (bands at 2924 cm^-1^, 2854 cm^-1^, 1737 cm^-1^ and 1713 cm^-1^) and iron oxides (peak at 528 and 470 cm^-1^) are present. In sample ASN4, also the bands associated to proteinaceous compounds have been detected, in particular the 1540 cm^-1^ peak due to amide II [16,17]. 

XRF analysis revealed the presence of zinc in all the examined samples but also of lead (Table 2). According to this result we can say that the white layer, visible in sample ASN2 and ASN5 cross sections was probably made of lead white. This painting was realized previously in respect to that made of zinc white. The presence of iron suggests the use of red, yellow and brown ochre. The green grains visible in the cross section of sample ASN2 are made of a copper based pigment. Sample ASN3, defined as gilding, contains zinc and copper suggesting the presence of brass powder used to imitate gold. Arsenic is a component of the alloy. The use of brass to imitate gold was particularly diffused during the 18^th^ century, especially to produce objects employed on the occasion of popular and traditional festivities [18]. 

**Table 2:** Results of XRF analysis expressed as count per second (cps) of the main elements found in the samples

**Sample**	**Ca**	**Fe**	**Zn**	**Pb**	**Ba**	**Sr**	**Cu**	**Mn**	**As**
ASN1 front	32	147	2738						
ASN1 back	65	146	373	313	tr				
ASN2 front	101	469	13678	544	tr	tr			
ASN2 back	954	80	301	594		188			
ASN2 red area	354	209	2616	542		143			
ASN2 green area	49	78	7604	344	55	75	130		
ASN3 front	101	170	759				536		205
ASN4 front	280	1748	3243	157	52	133		122	
ASN4 back	464	179	216	167					
ASN4 yellow area	115	285	486	270					
ASN5 front	332	133		597	110				

Calcium, with traces of strontium, is the main element of gypsum and calcite used to prepare the setting layers.

### Wood sample analysis

As referred in the background section, the carefully in situ observation of the Altar Machine allowed detect several damages on the wood elements like cracks, deformations, surface detachments and scratches, dust layers, colour modifications, and many holes caused by the insects. Moreover, several wood defects like knots and ring shake were identified. Wood deterioration was due to the manufacturing process, to the Altar Machine usage, and to the microclimate in the apsidal area of the church. The numerous galleries produced by the xylophagous insects weakened the wood, making the substitution of elements or the introduction of plugs necessary.

The identification of the wood species was undertaken because the diagnosis of the botanical species represents a great importance in the technological and historical artistic study of a wooden artefact due to possible implications relating to the conservation. In fact, every species exhibits peculiar characteristics that delineate the physical, mechanical and durability properties of the material as features of the xylem of the tree botanical species.

The species identification allowed to find six different kinds of wood that were used in relation to the technological characteristics of the altar elements. The results are summarized in Table 3.

**Table 3:** Botanical species found in the Altar Machine

**Sample**	**Location**	**Botanical species**
ASN6	Altar decoration	Poplar *Populus* spp.
ASN7	Pulley	Oak *Quercus* spp.
ASN8	Framework	Chestnut *Castanea sativa* Mill.
ASN9	Winch drum	Walnut *Juglans regia* L.
ASN10	Tilting table	Pine *Pinus* spp.
ASN11	Tabernacle	Fir *Picea abie* Karst.

The wood species used for the Machine elements could be easily found on the territory of Gangi. The availability and the cost of timber influenced the choice of the wood by local artisans [19]. The presence of *Picea abies* Karst in the tabernacle can be probably traced back to a restoration intervention. In fact, this alpine species, due to its excellent technological characteristics, has been widely marketed and often has been found in restored wooden structures in Sicily [20].

Chestnut wood was used in the back structural elements of the altar due to its excellent physical and mechanical properties in relation to the density. Moreover, the chestnut wood has a natural durability and it exhibits a moderate shrinking [21,22]. Chestnut wood has been used in Italy especially for roof beams and also for the outer surface of the doors due to the properties of its heartwood. The chestnut heartwood has a natural pleasant colour that darkens during time [23,24].

The walnut wood was found in the winch drum (cylindrical element on which the ropes are wrapped in order to raise the statue). The use of this species is related mainly to the low coefficient of shrinkage that ensures dimensional stability during the thermo hygrometric variations [21].

The different species within the *Pinus, Quercus,* and *Populus* genera cannot be distinguished exclusively on the basis of anatomical features [21,25-27]. Therefore the identification of botanical species was not obtained.

Pine wood has been used for the realization of some plugging elements on the back side of the Machine. These elements probably were movable, but today we are not able to understand their function.

Oak wood has been used to obtain the pulleys due to its high density and wear resistance [21].

The decoration elements are made of poplar, a wood species widely used in Italy for the creation of painted panels and other decorated works of art [21,28]. In fact, it is characterized by low density, easy seasoning and processing, and colour homogeneity that make it particularly suited to apply the painted layers, in spite of this wood could be easily attacked by xylophages.

### Microclimatic analysis

The microclimate campaign was carried out during the summer that can be considered the most critical period for the artefact. In fact, summer sultriness is particularly high during August in South Italy. Moreover, in August the Machine is moved on the occasion of religious celebrations and many people crowd the church influencing the microclimate.

It must be stressed that the monitoring campaign was short but, we think, significant and useful for the purpose of this work that is the overall study of a peculiar and interesting artefact with the hope that it could be restored and conserved in the most appropriate way.

**Figure 7:**
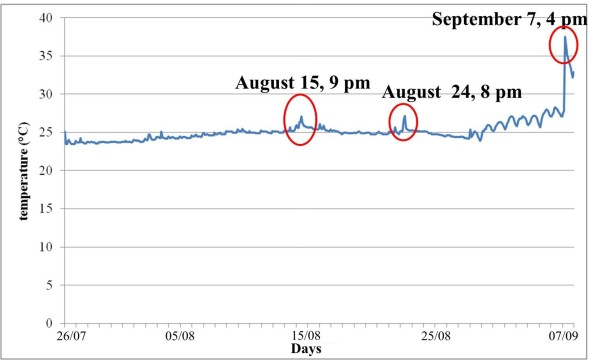
Graph of the temperature against time over the entire monitoring period.

**Figure 8:**
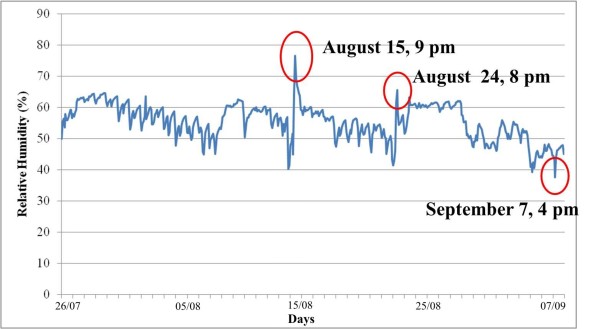
Graph of the relative humidity against time over the entire monitoring period.

By analysing the microclimatic data, two little peaks of temperature were registered in August (15^th^ and 24^th^) in the afternoon. They are due to the presence of many believers in the church (Figure 7). During August 15^th^, in fact, the Altar Machine is usually moved on the occasion of the *Assumption Day*. On August 24^th^ there was a celebration to ordain a priest and so again many believers crowded the church. In correspondence of the two peaks of temperatures, high values of relative humidity have been registered (Figure 8).

During the first days of September temperature values raised considerably and RH values decreased.

**Figure 9:**
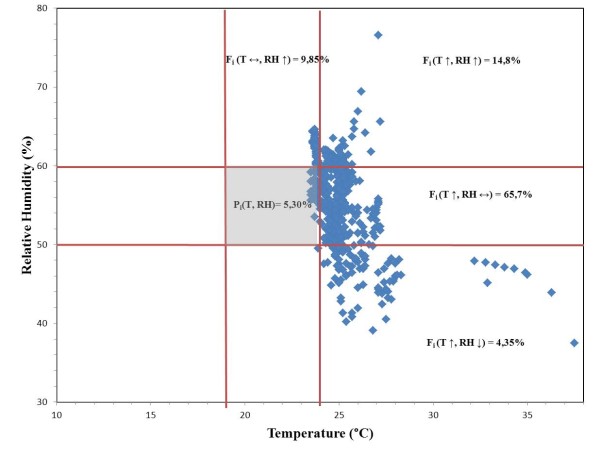
**T and RH performance and failure index in the tolerance matrix.** The grey area defines the tolerance area for painted wood, painted wood sculptures, painted panels, according to UNI10829.

The tolerance matrix (Figure 9) is particularly useful for the microclimate studies [14]. P_i_ represents the Performance Index that is the set of T and RH data falling within the tolerance range. F_i_ is the failure index that is the percentage of T and RH values falling outside the tolerance range. In general it is possible to assess that most data fall outside the tolerance area and only 5,30% is within the range. It is interesting to note that most data (71,0%) fall within the area where RH values are acceptable whereas T values are too high. But, T values appeared quite constant; on the contrary RH values fell almost always inside the s. i., but they often exhibited dangerous variations. 

The microclimatic data evaluation is summarized in Table 4.

**Table 4:** Thermo hygrometric data evaluation

**T range (max-min) °C**	**37.50 - 23.50**
RH range (max-min) %	76.60 - 37.50
Average T °C	25.20
Average RH %	55.43
Standard deviation T	1.61
Standard deviation RH	5.69
F_i_ % for T	84.85
F_i_ % for RH	29.00
Evaluation of risk for high values of T	high
Evaluation of risk for high values of RH	low
Evaluation of risk for quick variations of T	low
Evaluation of risk for quick variations of RH	high

In the heritage conservation perspective, the change in indoor climate needs to be interpreted in terms of impact on specific objects. The RH variations, controlled by temperature and moisture, are key factors in the conservation of the painted panels of the Altar Machine.

Wood swells and shrinks when adsorb or desorb moisture to reach a dynamic equilibrium with environmental relative humidity. Due to the anisotropic dimensional variation, to the moisture gradient in the thickness of the wood element, and to the different treatment of some surfaces, strain or failure can be observed [29]. Moreover, the wood’s internal damage can be cumulative and invisible micro fractures can precede the visible damage which appears only after the internal structure has progressively weakened or has accumulated sufficient stress [30]. Even a short heating episode can be dangerous especially when it is repeated before the wood has completely relaxed from the previous strain [31].

The several cracks observed in the Altar Machine generally follow the wood fibers and can be ascribed both to shrinkage/swelling caused by RH changes and to the movements of the Machine during its usage. A direct on-site monitoring of the mechanical damage in the wood elements could be performed by acoustic emission [32]. But in the case of the Altar Machine in Gangi it was not possible to perform this kind of analysis both for the high cost and also because the Machine is moved various times during the years.

We can assess that the microclimatic monitoring is a fundamental process to undertake in order to correctly plan the preservation of the artefacts. Often the microclimatic requirements for the artefact preservation diverge from the ones of the visitors in a museum or of the parishioners in a church, so it is not easy to define a balance between conflicting environment performance requirements [30].

## Conclusions

In this paper the results of the study of the Altar Machine in Gangi (Italy) are reported and discussed. The Altar Machine is a very peculiar artefact as regards its history, its demo-ethno-antropological significance and its technical execution. In spite of this, it was not much studied, like many other artefacts that are considered of minor importance within the art history. But, in our opinion the study of this kind of artefact could be important to know the traditions of a people; moreover the investigation on the original materials and the execution techniques, together with the monitoring of the microclimate, can supply information to correctly preserve the artefact.

The following conclusion can be pointed out:

 the characterization of the constitutive materials allowed to detect a lead based pigment used for the white painting on the front side of the Altar Machine that can be supposed the original pigment applied in the 18^th^ century. The surface zinc white layer, today visible, was probably applied during the 1908 restoration intervention. Iron and copper based pigments were also found in the painted layers of the Altar Machine. The presence of brass gilding further supports the dating of the Machine proposed by the inscription. The analysis of the wood elements allowed to find six different botanical species that were used in relation to the technological characteristics of the altar parts.

 The microclimate monitoring put in evidence that the temperature and relative humidity values are not always suitable to correctly preserve the artefact. In particular, RH% values exhibit dangerous variations, whereas T values are too high, 84,85% fall outside the safety interval; only 5,30% of the T-RH values fall within the safety intervals. High values of temperature can favour the microbiological attack, as can be observed directly on the wood elements of the Altar Machine. Fluctuating relative humidity values can cause mechanical stress on wood and, as a consequence, on the painted layers applied on its surface. The careful in situ investigation, also with the aid of a magnifier, confirmed an on-going climate induced damage to the Altar Machine that, associated to the deterioration caused by its usage, may have dramatic consequences on this unique and peculiar work of art.

## Competing interests

The authors declare that they have no competing interests.

## Authors’ contributions

ALM carried out the characterization of the wood species, the literature research and the general organization of the paper. MM coordinated the study and modified the text. CP performed the analysis of the painting materials, the microclimate monitoring and the data treatment. MS carried out the technical survey, the historical and archive research on the Altar Machine. All authors read and approved the final manuscript.
